# Venlafaxine treatment reduces the deficit of executive control of attention in patients with major depressive disorder

**DOI:** 10.1038/srep28028

**Published:** 2016-06-16

**Authors:** Yanghua Tian, Jing Du, Alfredo Spagna, Melissa-Ann Mackie, Xiaosi Gu, Yi Dong, Jin Fan, Kai Wang

**Affiliations:** 1 Department of Neurology, the First Hospital of Anhui Medical University, Hefei, Anhui Province, China; 2 Department of Neurology, the Second Hospital of Anhui Medical University, Hefei, Anhui Province, China; 3 Department of Psychology, Queens College, the City University of New York, Flushing, NY, USA; 4 Center for BrainHealth, School of Behavioral and Brain Sciences, the University of Texas at Dallas, Dallas, TX, 75235, USA; 5 Anhui Mental Health Center, Hefei, Anhui Province, China; 6 Department of Psychiatry, Icahn School of Medicine at Mount Sinai, New York, NY, USA; 7 Department of Neuroscience, Icahn School of Medicine at Mount Sinai, New York, NY, USA

## Abstract

Attention plays an essential role in supporting other cognitive functions and behavior, and disturbance of attention is one of the most common symptoms in major depressive disorder (MDD). Although treatment with venlafaxine for MDD symptoms has been shown to reduce deficits in cognition and emotion regulation, it remains unclear whether venlafaxine improves specific attentional functions. We used the Attention Network Test to measure the attentional functions of alerting, orienting, and executive control before and after treatment with venlafaxine in patients with MDD compared to untreated healthy controls. Before treatment, the MDD group showed a selective impairment in alerting and executive control of attention, while there were no significant group differences in the orienting function. The interaction between group and session was significant for executive control, and after treatment with venlafaxine, the performance of the MDD group on executive control of attention was not significantly different from that of controls. Reported symptoms of MDD were also significantly reduced after treatment with venlafaxine. These results demonstrate that treatment with venlafaxine selectively normalizes the executive control function of attention in addition to improving clinical symptoms in MDD.

Major depressive disorder (MDD) is the most common type of psychiatric disorder, with lifetime prevalence estimates of more than 3.5% in China^1^. In addition to mood symptoms, it is often associated with deficits in attention^2^,^3^,^4^, executive functions^4^,^5^,^6^, and processing speed^5^,^7^. These are considered to be primary features of the disorder and have a negative impact on functional and social ability^8^,^9^,^10^. Attention plays an essential role in supporting other cognitive functions and behavior, and its disturbance is one of the most common symptoms in MDD, with frequent complaints of difficulty maintaining concentration^10^. A wealth of studies have provided empirical evidence for these attention deficits, such as in the alerting^2^,^3^,^11^ and orienting^11^ functions of attention, although some controversy still exists^4^,^11^. However, whether current pharmacological interventions are effective in the treatment of these deficits is unclear. Hence, a comprehensive assessment of treatment outcomes should investigate the change in cognitive functions, such as attention, together with changes in the clinical symptoms.

Attention can be conceptualized as three separable functions of alerting, orienting, and executive control, supported by corresponding brain networks and neurotransmitter systems^12^,^13^. Alerting contributes to the maintenance of readiness and has been associated with activation in the thalamus, and frontal and parietal cortical regions^14^, and with the cortical distribution of the norepinephrine (NE) neurotransmitter system^15^. Orienting is responsible for selecting and moving attention to stimuli, and is supported by frontal and parietal regions and the acetylcholine (Ach) neurotransmitter system^16^,^17^. Executive control is involved in resolving conflicts and coordinating among thoughts and actions^18^,^19^, and often activates areas in the frontoparietal network (FPN) including the anterior cingulate cortex (ACC) and other frontoparietal regions^14^,^20^, and is modulated by the mesocortical dopamine (DA) system^21^. The efficiency of these attentional functions can be measured using the Attention Network Test (ANT)^13^. Using this paradigm, selective and multiple attentional function impairments have been found to be associated with different psychiatric disorders^22^,^23^. Although previous studies have examined specific attentional function deficits in MDD using other tasks^2^,^3^,^4^,^24^, the selective impairment of attention in MDD and treatment effects on attention are still unclear.

Deficits in attentional systems may be linked to dysfunction in specific neurotransmitter systems that have been associated with MDD. Specifically, dysfunctions in serotonin (5-HT), NE, and DA systems have been consistently associated with MDD^25^ and current antidepressant treatments act on these neurotransmitters to reduce the clinical symptoms. Venlafaxine is an antidepressant in a group of drugs called serotonin-norepinephrine reuptake inhibitors (SNRI), and is recognized as a safe, rapidly effective, and widely used antidepressant^26^,^27^. Its main mechanism of action is the inhibition of 5-HT reuptake, with dose-dependent inhibition of NE reuptake^28^, and weak DA reuptake inhibition^29^. Furthermore, there is evidence that venlafaxine might also increase dopamine levels in striatum, hippocampus, and frontal brain regions^28^,^30^,^31^,^32^. Given that venlafaxine acts on neurotransmitters systems relevant to attention, and the persistence of attentional deficits in patients with MDD^33^, it is important to clarify whether treatment with this antidepressant also treats deficits in the attentional functions.

The present study examined the effects of treatment with venlafaxine on the attentional deficits associated with MDD. Because NE and DA are implicated in the neurobiology of MDD, we hypothesized that patients with MDD would show impairment in the alerting and executive control functions. Importantly, because venlafaxine acts upon these neurotransmitters, we also hypothesized that the treatment with venlafaxine would reduce these attentional deficits.

## Results

### Clinical symptoms and treatment effects

**Figure 1:**
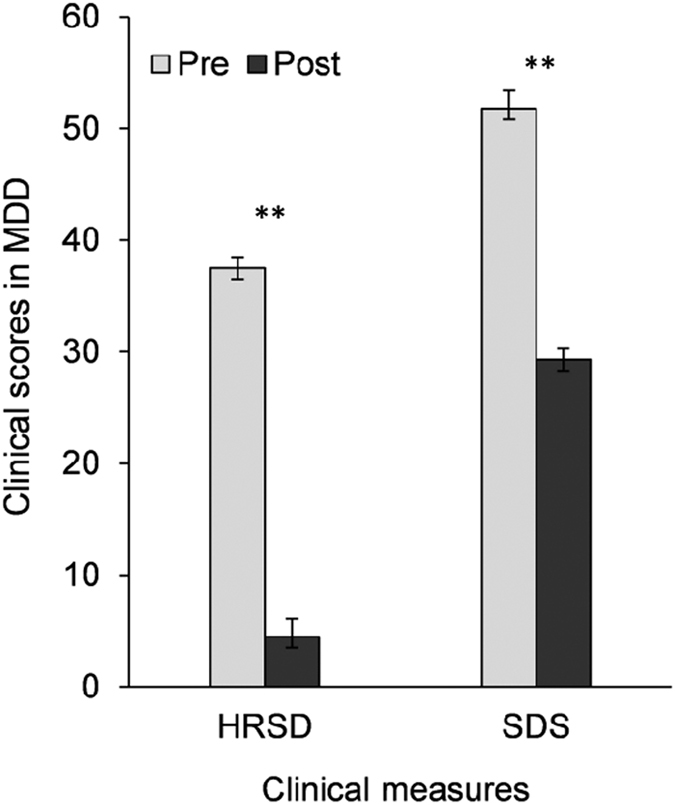
Clinical symptom scores at pre- and post-test in MDD patients treated with venlafaxine. Note: **p < 0.01.

There were no significant differences between the MDD and HC groups in age, education, or MMSE scores (see Table 1). In the MDD group, the 24-item Hamilton Rating Scale for Depression (HRSD)^34^ scores decreased significantly from the pre-test (37.5 ± 5.8) to post-test session (4.5 ± 5.6), (*t*_(33)_ = 25.4, *p* < 0.001). The Self-Rating Depression Scale (SDS)^35^ scores also decreased significantly from pre-test (51.8 ± 9.4) to post-test (29.3 ± 5.6), (*t*_(33)_ = 12.1, *p* < 0.001). See Fig. 1 for the treatment effects of clinical symptoms.

### Overall reaction time and error rate

**Figure 2:**
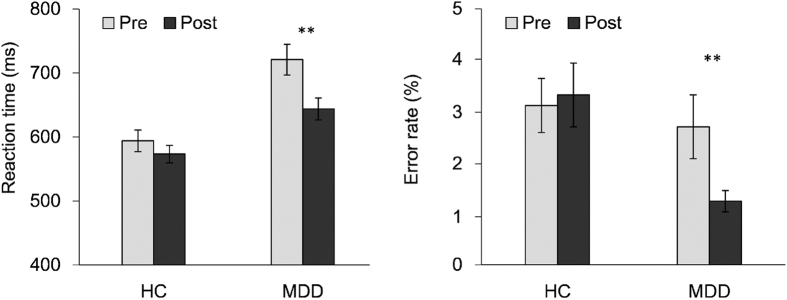
Overall reaction time and error rate for MDD and HC groups in the pre- and post-test sessions. Patients with MDD showed a significantly slower responding speed than controls in pre-test session. After treatment with venlafaxine, there was a significant improvement in response time in the MDD group. Note: **p < 0.01.

Table 2 and Fig. 2 show the overall reaction time (RT) and error rate (ER) group differences. Trials with 3 standard deviations (SD) above the mean RT were considered outliers and excluded from further analysis. For the overall RT, the main effect of Group was significant (*F*_(1,62)_ = 15.4, *p* < 0.001) indicating that the MDD group (683 ± 120 ms) responded significantly slower than the HC group (584 ± 84 ms). The main effect of Session was significant (*F*_(1,62)_ = 32, *p* < 0.001) indicating that RT was reduced from pre-test (661 ± 136 ms) to post-test (611 ± 95 ms). The Group by Session interaction was significant (*F*_(1,62)_ = 10.7, *p* < 0.05). Simple comparisons revealed that in the MDD group, RT decreased significantly from pre-test (721 ± 141 ms) to post-test session (644 ± 100 ms) (*F*_(1,62)_ = 42.7, *p* < 0.01), while the difference in RT between the two sessions (594 ± 91 ms and 573 ± 73 ms, respectively) in the HC group was not significant (*F*_(1,62)_ = 2.7, *p* = 0.10).

For the overall ER, the main effect of Group was significant (*F*_(1,62)_ = 4.1, *p* < 0.05), indicating that the HC group (3.1 ± 2.9%) made more errors than the MDD group (1.9 ± 2.3%). The main effect of Session was not significant (*F*_(1,62)_ = 3.5, *p* = 0.07). The Group by Session interaction was significant (*F*_(1,62)_ = 6.4, *p* < 0.05). Simple comparisons revealed that the ER was significantly reduced from the pre-test session (2.6 ± 3.1%) to the post-test session (1.2 ± 1.3%) (*F*_(1,62)_ = 10.3, *p* < 0.01) in the MDD group, while the difference in the ER between the two sessions (3.0 ± 2.8% and 3.2 ± 3.1%, respectively) in the HC group was not significant (*F* < 1).

### Attentional deficits and treatment effects

**Figure 3:**
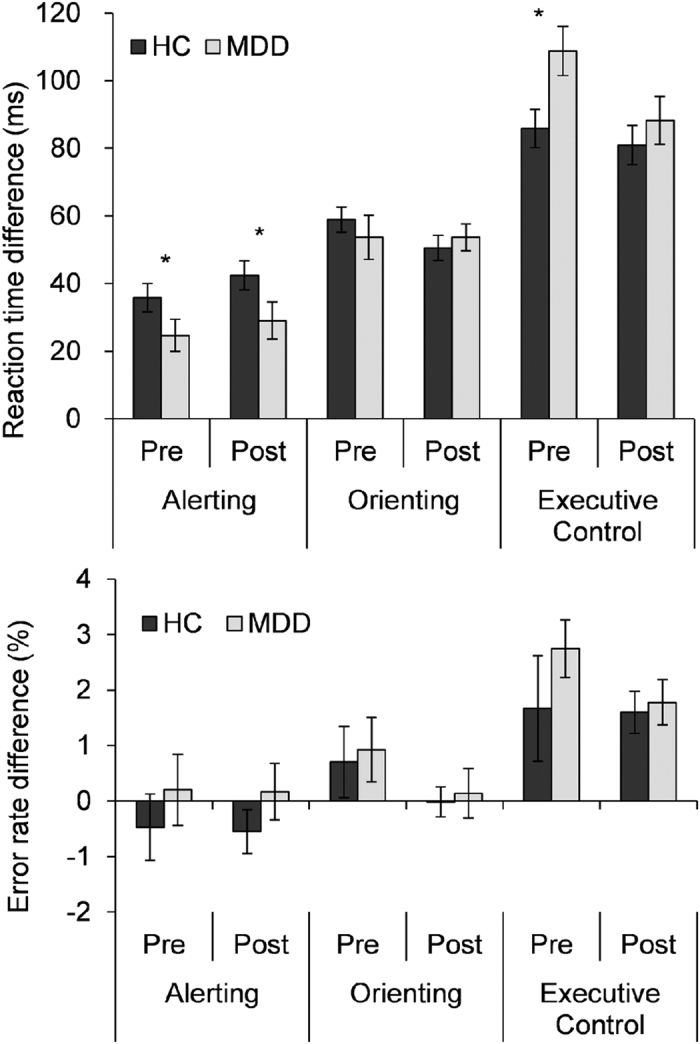
The attentional effects for the MDD group and for to the HC group in the pre- and post-test sessions. In the pre-test session, the MDD group showed a selective impairment in alerting and executive control of attention in reaction time. In the post-test session, the performance of the MDD group on executive control of attention was not significantly different from that of HC in reaction time. Note: *p < 0.05.

Table 2 and Fig. 3 show the attentional effects for both groups within the two sessions.

#### The alerting effect

For RT, the main effect of Group was significant (*F*_(1,62)_ = 12.3, *p* < 0.05), indicating a smaller alerting effect in the MDD group (27 ± 30 ms) compared to the HC group (39 ± 23 ms). The main effect of Session was not significant (*F*_(1,62)_ = 1.7, *p* = 0.19). The Group by Session interaction was not significant (*F*_(1,62)_ = 1.8, *p* = 0.19). For ER, the main effects of Group (*F*_(1,62)_ = 1.4, *p* = 0.24), and Session (*F* < 1), and the Group by Session interaction (F < 1) were not significant.

#### The orienting effect

For RT, the main effects of Group and Session (*Fs* < 1) and the Group by Session interaction effect (*F*_(1,62) _= 1.1, *p*_ _= 0.29) were not significant. For ER, the main effects of Group (*F* < 1) and Session (*F*_(1,62) _= 2.0, *p*_ _= 0.15), and the Group by Session interaction (*F* < 1) were not significant.

#### The executive control effect

For the RT, the main effect of Group was not significant (*F*_(1,62) _= 3.1, *p*_ _= 0.09). The main effect of Session was significant (*F*_(1,62) _= 13.2, *p* < 0.01), indicating a greater conflict effect in the pre-test (98 ± 38 ms) compared to post-test (post 85 ± 37 ms). The Group by Session interaction was significant (*F*_(1,62) _= 4.9, *p* < 0.05). Simple comparisons indicated that the executive control effect was significantly reduced from pre-test session (109 ± 43 ms) to post-test session (88 ± 41 ms) in the MDD group (*F*_(1,62) _= 18.3, *p* < 0.01), while the difference in the conflict effect between the two sessions was not significant for the HC group (*F* < 1). The post-test difference in the conflict effect between the HC and the MDD groups was not significant (*F*_(1,62)_ = .84; *p* = .36). Bayesian t test for accepting or rejecting the null hypothesis (http://pcl.missouri.edu/bf-two-sample) favored the null. For ER, the main effects of Group (*F* < 1), Session (*F* < 1), and the Group by Session interaction (*F* < 1) were not significant.

#### Correlations between attentional functions and clinical scores

There were no significant correlations between attentional functions and symptom scores of the HRSD and SDS neither at pre-test, nor between the attentional effect change and symptom change scores at post-test.

## Discussion

This study demonstrated slower overall response speed in the MDD group, as well as pre-treatment attentional deficits in the alerting and executive control functions of attention. This result is consistent with other studies showing that depression is associated with deficits in executive control^4^,^5^,^6^,^36^. The executive control of attention has been extensively related to the activity of several frontal and parietal areas (FPN^37^), and in particular to the activation of the ACC, which depends on the mesocortical DA system^38^,^39^,^40^. Hypofunction and abnormal structure in ACC have been shown to be associated with major depression^41^,^42^. Therefore, the impairment of executive control function in MDD may be caused by dysfunction of this region within the FPN.

Furthermore, venlafaxine treatment selectively improved the executive control component of attention. This observed selective treatment effect may be due to direct and indirect effects of this medication on the DA system which improved the efficiency of this function within the patient group. Previous studies have demonstrated that venlafaxine increases DA level in frontal lobe and limbic system^30^,^31^,^32^. Furthermore, there is abundant physiological evidence for complex modulation and interactions between 5-HT and DA systems within the frontal lobes. 5-HT has been shown to have an inhibitory effect on DA neurotransmission^43^,^44^, and a reduction in available 5-HT, as implicated in the neurobiology of MDD, may result in release from inhibition of the DA system, resulting in impulsivity, a hallmark of deficient executive control. Consequently, increasing the availability of 5-HT within frontal cortex via SNRI treatment may account for improvement of the executive control of attention^45^,^46^. However, further investigations that directly compare drugs designed to target different neurotransmitter systems are needed in order to draw strong conclusions about the specificity of these mechanisms.

The improvement of executive control of attention is not trivial. A previous study demonstrated that this attentional function contributes significantly to the implementation of cognitive control, which is necessary for executing high-level cognitive functions^47^. The efficiency of cognitive control and executive functions is directly related to functional outcomes in MDD^48^,^49^. Furthermore, cognitive control is involved in emotion regulation^50^ and the suppression of dysfunctional thoughts^51^, which are common in MDD. Consequently, treatment of this attention component may result in improvement in cognitive control, emotion regulation, and in overall daily functioning.

Based on the deficient NE neurotransmission associated with MDD, we also predicted that the patient group would show a deficit of the alerting function (which is related to the NE system^52^), and that this deficit would be reduced by venlafaxine. The reduced alerting effect observed in the patient group suggests a lower level of readiness to respond to external stimuli^2^. However, we did not find evidence of a treatment effect with venlafaxine on the alerting deficit. It has been noted that the inhibition of reuptake of NE typically occurs at dosage greater than 150 mg/day^28^,^53^; 150 mg/day was the maximum dose used in this study and therefore may not have been high enough to directly act on the NE system. Given that typical maximum clinical doses can be as high as 375 mg per day, and previous evidence indicating that significant effects on the noradrenergic system are achieved only with high doses of venlafaxine^54^, it is possible that higher dosages than were used in this study might have a treatment effect on the alerting function, though higher doses tend to increase the likelihood of adverse side effects^55^.

The prediction that there would be no deficit in the orienting of attention associated with MDD, as shown in previous studies^2^,^4^, was confirmed also by our results. Furthermore, venlafaxine does not act on the cholinergic system^28^, and we did not expect any change in the orienting function due to the treatment. Previous studies have demonstrated that both the depletion of 5-HT and NE-blocking drugs, the two mechanisms by which venlafaxine exerts its effect, have no influence on the orienting performance^56^,^57^.

Although cognitive impairments are nowadays often associated with major depression^5^, the relationship between attentional deficits and clinical symptoms of MDD is still under debate. For example, there is some evidence showing that the cognitive deficits and clinical symptoms may be due to abnormalities of cortical and subcortical regions, however, the cognitive impairment seems to be more durable than the clinical symptoms^58^. In the current study, although the treatment with venlafaxine reduced the deficit in the executive control function together with effectively reducing the clinical symptomatology, the changes in the two measures were not correlated, which may suggest that the attentional deficits and symptom severity may arise from independent mechanisms.

There are some limitations to this study that may restrict the strength of the conclusions. The sample size in this study was relatively limited, and a larger study would allow for stronger conclusions about the attentional deficits and treatment effects. Furthermore, because all patients received treatment with venlafaxine only, we were not able to compare the effects of different types of antidepressants on attentional functions. Such a comparison could help to further clarify the role of intervening at the level of neurotransmission to improve the cognitive and clinical symptoms of MDD. Future studies may also aim to increase the treatment duration (longer than the six weeks in this study) to determine the optimal treatment length for maximum gain in attentional improvement.

In conclusion, we found that there were deficits in the alerting and executive control of attention in MDD, and showed that venlafaxine selectively improved the executive control of attention. There was no evidence for deficits in the orienting function. Antidepressants that improve cognitive function in addition to clinical symptoms have great potential to reduce the functional impairment associated with MDD.

## Methods

### Participants

Fifty-three patients with MDD were recruited from Anhui Mental Health Center affiliated with Anhui Medical University, China. Diagnosis of MDD was by consensus of two independent psychiatrists using the Structured Clinical and Interview for DSM-IV. MDD participants were drug naïve or drug free for at least 3 months prior to the study, and only patients eligible for venlafaxine treatment were recruited and were monitored for dose titration and adverse side effects. The 24-item HRSD and SDS were used to measure the severity of clinical symptoms. The Mini-Mental State Examination (MMSE)^59^ was administered to all participants and only those who scored higher than 27 were included in order to exclude mild cognitive impairment and dementia. Patients with a history of brain tumor, stroke, or other neurological disease that could interrupt brain function were excluded. Four patients showing intolerance to the treatment with venlafaxine were excluded from this study and received an alternative antidepressant treatment. In total, 19 patients discontinued their participation in this study. The final MDD sample consisted of thirty-four patients (10 males and 24 females; mean age = 36 ± 13 years; average years of education = 11 ± 4 years).

Thirty healthy controls (HC; 11 males and 19 female) were recruited (mean age = 34 ± 12.2 years; average years of education = 11 ± 4 years). HC participants were evaluated by staff psychiatrists, and individuals with history of neurological, psychiatric, or systemic medical disorders were not included. All participants had normal or corrected to normal vision and gave written informed consent. The ethical committee of Anhui Medical University approved this study, and methods and procedures of this study were in accordance with the approved guidelines.

### Attention Network Test

**Figure 4:**
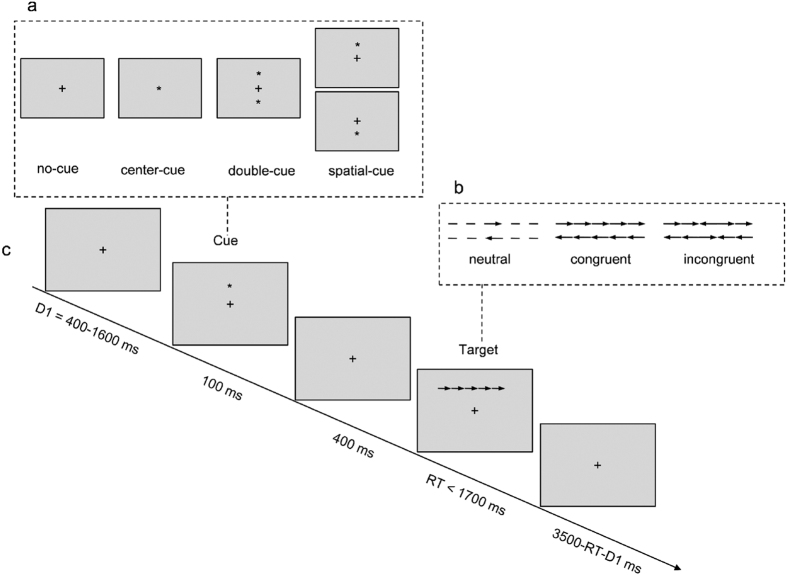
Experimental procedure: (**a**) the four cue conditions; (**b**) the three target conditions (six target types) used in the present experiment; and (**c**) an example of the procedure. In this task, participants made responses to indicate the direction of a central arrow (left or right).

Figure 4 illustrates the stimuli and sequence of events in the ANT. Stimuli consisted of a row of five visually presented horizontal black lines, with arrowheads pointing leftward or rightward, against a gray background. The target was a left- or right-pointing arrowhead in the center, flanked on either side by two arrows pointing in the same direction (congruent condition), or in the opposite direction (incongruent condition), or by horizontal lines (neutral condition). A single arrow or line extended 0.55° of the visual angle and the contours of adjacent arrows or lines were separated by 0.06° of the visual angle. The row of five stimuli was presented at 1.06° either above or below the central fixation cross. Participants were asked to identify the direction of the central arrow by pressing one computer mouse button for the left direction and a second button for the right direction. Cues consisted of a 100 ms asterisk presented 400 ms before the target. There were four cue conditions: (1) no-cue, in which the central fixation cross remained present and unchanged; (2) central-cue, which appeared at the central fixation point; (3) double-cue, in which cues were presented on the two possible target locations simultaneously; and (4) spatial-cue, in which the cue was presented at the location of the upcoming target. The task consisted of a 24-trial practice block and three experimental blocks of trials. Each experimental block consisted of 96 trials (48 conditions: 4 warning levels × 2 target locations × 2 target directions × 3 congruency conditions, with 2 repetitions). The presentation of trials was randomized. Participants were instructed to fixate at a centrally located cross throughout the task, and to respond as quickly and accurately as possible.

Effects for each attentional function were calculated based on the RT and ER data. The alerting effect was calculated by subtracting the mean RT of the double cue condition from the mean RT of the no cue condition. The orienting effect was calculated by subtracting the mean RT of the spatial cue condition from the mean RT of the center cue condition. For the ER computations of alerting and orienting, the subtractions were reversed to yield positive effect scores. The executive control effect was calculated by subtracting the mean RT (or ER) of congruent conditions from the mean RT (or ER) of incongruent conditions. The calculations of attentional effects are described in detail in a previous publication^13^.

### Procedure

In the pre-test session, participants from both groups completed the MMSE, and the ANT. The MDD group also completed the HRSD and SDS. MDD patients were then treated with venlafaxine with a starting dose of 75 mg, gradually increased up to 75–150 mg daily. At the 6-week time point (post-test session) the MDD group then completed the ANT, HRSD and SDS again. During the six-week period, patients did not receive any other treatment. The untreated HC group was also post-tested on the ANT after a 6-week interval.

### Data Analysis

Mixed factorial analyses of variance with Group (HC, MDD) as the between-subjects factor and Session (pre-test, post-test) as the within-subjects factor were performed on each attentional effect in both RT and ER. Simple comparisons were used to further analyze significant interaction effects. Spearman correlation analyses were conducted between attentional effects and pre-test clinical symptoms, and between changes (pre-test minus post-test) in the attentional effects and changes in the clinical symptoms after venlafaxine treatment, and a corrected critical α value of *p* < 0.01 was used.

## Additional Information

**How to cite this article**: Tian, Y. *et al*. Venlafaxine treatment reduces the deficit of executive control of attention in patients with major depressive disorder. *Sci. Rep.* **6**, 28028; doi: 10.1038/srep28028 (2016).

## Figures and Tables

**Table 1:** Demographic data of patients with MDD and healthy controls (Mean ± SD).

	MDD (n = 34)	HC (n = 30)	Test	*p*
Sex (Female/Male)	24/10	19/11	*χ*^*2*^ = 0.38	0.54
Age in years	36.1 ± 13.3	34.2 ± 12.2	*t* = 0.58	0.56
Education in years	10.6 ± 3.9	10.8 ± 3.8	*t* = 0.29	0.78
MMSE	29.3 ± 1.2	29.5 ± 0.8	*t* = 0.68	0.45

Note: MMSE = Mini-mental state examination.

**Table 2:** Attention network scores for Reaction Time (SD), in ms, and Error Rate (SD), in percent, of MDD and HC groups.

	MDD (n = 34)		HC (n = 30)	
	Pre-test	Post-test	Pre-test	Post-test
Reaction Time				
Overall	721 (141)	644 (100)	594 (91)	573 (75)
Alerting	25 (28)	29 (32)	36 (23)	42 (23)
Orienting	54 (38)	54 (23)	59 (21)	51 (21)
Executive control	109 (43)	88 (41)	86 (31)	81 (32)
Error Rate				
Overall	2.6 (3.3)	1.2 (1.3)	3.0 (2.8)	3.2 (3.1)
Alerting	0.2 (3.5)	0.2 (2.4)	−0.5 (3.5)	−0.6 (2.8)
Orienting	0.9 (3.7)	0.1 (1.6)	0.7 (3.2)	−0.02 (2.5)
Executive control	2.8 (5.6)	1.8 (2.2)	1.7 (2.9)	1.6 (2.2)
